# A novel technique to repair orbital roof defects: irradiated homologous cadaveric rib (Tutoplast ®) graft in a recurrent frontal sinus ossifying fibroma

**DOI:** 10.1093/jscr/rjac535

**Published:** 2022-11-23

**Authors:** Vikas Acharya, Jimmy Ng, Annakan Navaratnam, Catherine Rennie, Peter Clarke

**Affiliations:** Department of ENT Surgery, Charing Cross Hospital, Imperial College Healthcare NHS Trust, London W6 RF, UK; Department of ENT Surgery, Charing Cross Hospital, Imperial College Healthcare NHS Trust, London W6 RF, UK; Department of ENT Surgery, Charing Cross Hospital, Imperial College Healthcare NHS Trust, London W6 RF, UK; Department of ENT Surgery, Charing Cross Hospital, Imperial College Healthcare NHS Trust, London W6 RF, UK; Department of ENT Surgery, Charing Cross Hospital, Imperial College Healthcare NHS Trust, London W6 RF, UK

**Keywords:** orbital roof defects, orbital repair, Tutoplast, ossifying fibroma

## Abstract

Ossifying fibroma in the fronto-ethmoidal sinuses is a rare, benign condition. In symptomatic cases, surgical excision is often undertaken and bony defects may be repaired using alloplastic grafts. We present a novel method of repairing an orbital roof defect using irradiated homologous cadaveric rib (Tutoplast ®) graft, overlaid with a pericranial flap. The patient made an excellent recovery, concluding that it is a viable and safe option with lower morbidity.

## INTRODUCTION

Ossifying fibroma (OF) in the mandible was first described by Montgomery in 1927 [1]. Classified as fibro-osseous tumour along with fibrous dysplasia and osteoma, OF is benign but can be clinically aggressive as it grows to pressurize neighbouring structures [2, 3]. In the paranasal sinuses, vast majority are found in the fronto-ethmoidal region [4].

Surgical excision is commonly performed in symptomatic cases and trans-nasal endoscopic approach is generally recommended, but limited to cases where complete excision is achievable, thereby necessitating an external approach or a combination of both in lesions that originate or extend to the lateral parts of frontal sinuses. In these circumstances, reconstruction is often undertaken [3, 5].

Repair of orbital roof following resection of frontal sinus OF is rarely described in the literature. We present a novel method technique whereby homologous cadaveric rib graft was used to repair orbital roof defect following combined approach resection.

## CASE REPORT

A 23-year-old patient was diagnosed with signs and symptoms of a right orbital abscess, optic nerve compression causing red desaturation and ocular dystopia, which was initially treated with an access septoplasty and endoscopic sinus surgery including clearance of the frontal recess. She was found to have an OF in the right frontal sinus, which was subsequently removed through a modified Lothrop procedure in 2010. A further extended modified Lothrop and an external approach to treat new bone formation was undertaken in 2013.

At a follow-up clinic 5 years after her last surgery, she complained of deterioration in vision and worsening facial asymmetry. On examination, there was marked right sided proptosis, ocular dystopia and hypoglobus, a significant horizontal diplopia and global restriction in all gazes was also noted. She had a visual acuity of 6/18 on the right, 6/6 on the left, with a slight relative afferent pupillary delay; colour vision was normal. Nasal endoscopy revealed a clear nasal cavity and patent frontal sinuses with no crusting visible.

**Figure 1:**
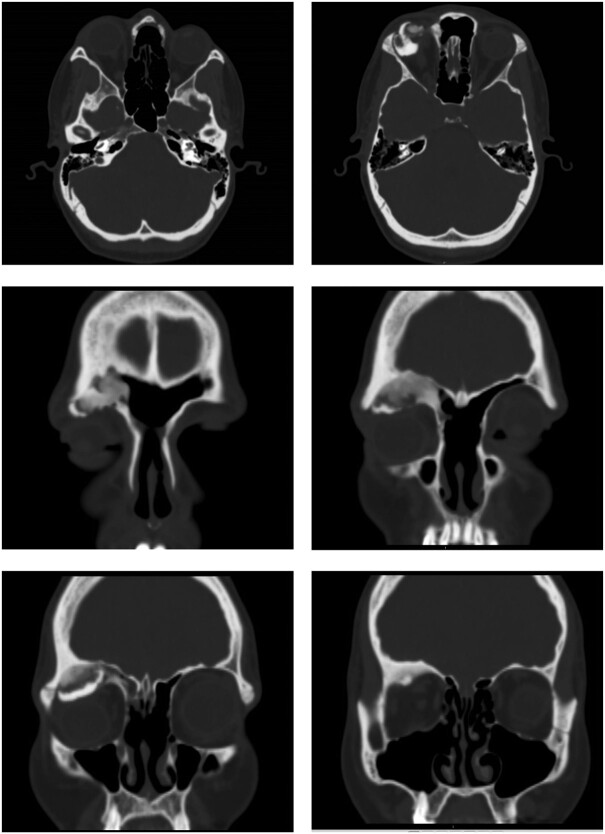
Ossifying fibroma in the right frontal sinus involving anterior and lateral frontal sinus walls, and orbital floor.

Computed tomography (CT) of the sinuses revealed proptosis, downward displacement and flattening of the upper surface of globe because of a recurrent right frontal sinus OF (Fig. 1).

**Figure 2:**
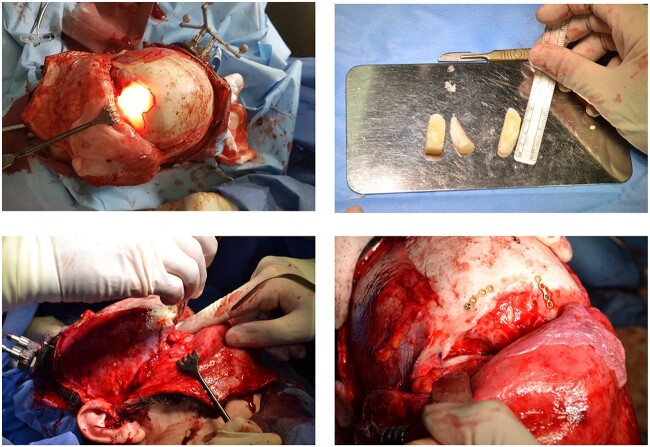
Combined endoscopic and bicoronal approach with the cadaveric rib graft cut to size to fit and repair the orbital floor defect.

She underwent an external bicoronal approach, frontal sinusotomy with an osteoplastic flap. The frontal sinus was opened and the OF shelled out, including the orbital roof connection. A 3 cm × 2.5 cm defect in the orbital roof, was closed with an irradiated cadaveric rib (Tutoplast ®) graft fashioned to size (Fig. 2). This was overlaid with a pericranial flap. An osteoplastic flap was used to close the frontal sinusotomy, secured with screws.

A histopathological study found areas of fibrous stroma with trabecular arrangement of immature bone without peripheral osteoblasts, as well as areas of myxoid stroma containing irregular trabeculae of immature bone with a rim of surface osteoblasts. The diagnosis was confirmed as a recurrent OF.

**Figure 3:**
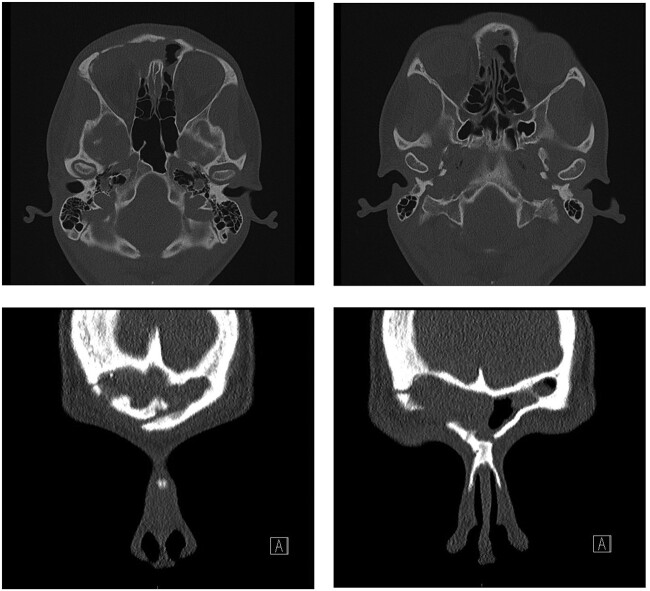
Repeat CT scan at 4 months after surgery, demonstrating no residual disease and radiological improvement of proptosis.

Post-operatively, the patient recovered well with no complications. Examination of the eye four weeks after surgery showed significant improvement—visual acuity was 6/6 on the right, 6/5 on the left. The visual acuity improved to 6/6 on both sides 10 months after surgery; however, there was a residual hypotropia. A repeat CT scan at 4 months post-surgery displayed no bulky residual disease and improvement of proptosis (Fig. 3).

## DISCUSSION

OF is a benign fibro-osseous lesion that can be further classified histo-pathologically into three variants: cemento-OF, juvenile active OF and aggressive psammomatoid OF [2, 6]. A systematic review in 2013 by Manes et al. found no distinct clinical significance between the histopathologic variants [6]. The lesion is often found incidentally but if symptomatic, cases originating from paranasal sinuses are frequently a consequence of ‘mass effect’ [3, 4, 7]. It is speculated that the initial occurrence or growth of the lesion occurs during childhood but the age at diagnosis is predominantly during the second and third decades of life [4].

An endoscopic approach is commonly regarded as the surgical treatment of choice; however, the central tenet remains incomplete excision of the disease, as recurrence may be more difficult to treat, given a number of factors—altered anatomy, scarring and distortion of structures and tissue planes [3, 4]. Another consideration in surgical resection is the avoidance of cerebrospinal fluid leaks, damage to the optic nerve or cavernous carotid injury [6]. In our case, the patient had undergone a modified Lothrop at the age of 15; therefore, considerable conformational changes of the skull would have occurred. Additionally, her recurrent tumour involved the orbital floor, anterior, lateral and frontal walls of the sinus. Balancing the risks of potential complications, a combined approach coupled with image-guidance to ascertain the depth of surgical field was employed in treating the recurrence in this case.

Schick et al. described good aesthetic and functional outcomes after utilizing outer table grafts to repair the anterior frontal sinus wall [5]; however, orbital roof involvement was not discussed. Our case presents a clinical challenge as it is a recurrent disease involving the orbital roof, with previous modified Lothrop resection altering the anatomy, and where an external approach is required to achieve complete resection.

Repairs are commonly performed for fractures following high-impact trauma using titanium plating or micromesh with screws to fixate the material as required, however bone and cartilage autografts may also be used [8, 9]. Despite the commonly described approaches of utilizing titanium mesh or autografts, we found that using an irradiated homologous cadaveric rib cartilage graft negates the need for autologous harvest, which is associated with morbidities and pain. As the defect in the orbital roof was sizably large (2.5 × 3 cm), the homologous cadaveric graft (Tutoplast ®) was cut to fit the defect and enforced with an overlay of pericranium. In a large-scale retrospective review of the use of 1025 irradiated homologous costal cartilage grafts in 357 patients, the overall complication rate was 3.25% (inclusive of warping, infection, infective resorption, non-infective resorption, mobility and extrusion) [10]. Although there are no similar data to evidence the use of these grafts in orbital floor repair, it is not unreasonable to expect a lower risk of complication as the surgical field was sterile and would continue to be so, unlike in the nasal cavities. Additionally, our patient had not reported any recognized adverse surgical outcomes (e.g. infection, cerebrospinal fluid leak, bleeding, graft failure) associated with either the craniofacial or endoscopic sinus surgeries on this sitting [3, 6]. Donor site complications associated with the use of autologous costal cartilage can include pneumothorax, pleural tear, infection, seroma, severe donor site pain and scarring [11].

To our knowledge, this is the first case in the literature where irradiated homologous cadaveric rib (Tutoplast ®) graft has been used to repair an orbital roof defect following combined approach resection of a recurrent OF. It is shown to be safe without the need to harvest rib or bone from the patient, thereby negating the associated potential complications and pain.
